# Flaxseed Fiber-Structured Nanoemulgels for Salad Dressing Applications: Processing and Stability

**DOI:** 10.3390/gels11090678

**Published:** 2025-08-24

**Authors:** María-Carmen Alfaro-Rodríguez, Fátima Vela, María-Carmen García-González, José Muñoz

**Affiliations:** Departamento de Ingeniería Química, Escuela Politécnica Superior, Universidad de Sevilla, C/Virgen de África, 7, E41011 Sevilla, Spain; fvela1@us.es (F.V.); mcgarcia@us.es (M.-C.G.-G.); jmunoz@us.es (J.M.)

**Keywords:** nanoemulgel, salad dressing, flaxseed fiber, microfluidization, rheology, temperature, stability

## Abstract

This study aimed to investigate the production of nanoemulgels structured with flaxseed fiber, designed to simulate salad dressings. For this purpose, the influence of microfluidizer passes (from one to four) on physicochemical and rheological properties was determined, followed by an assessment of thermal behavior. Rotor–stator homogenization followed by microfluidization were employed to produce nanoemulgels, which were characterized using laser diffraction, multiple light scattering, and rheological measurements. The resulting systems exhibited monomodal particle size distributions with mean diameters below 220 nm. Increasing the number of microfluidizer passes from one to four led to slight reductions in particle size, although they were not statistically significant. The formulation with two passes demonstrated superior physical stability during aging studies. Rheological evaluation indicated enhanced gel-like behavior with up to three passes, whereas excessive energy input (four passes) slightly compromised structural integrity. The linear viscoelastic region decreased notably after the first pass but remained relatively stable thereafter. The two-pass nanoemulgel, identified as the optimal formulation, was further tested for thermal stability. Temperature increases (5–20 °C) led to minor decreases in viscosity and firmness, yet the structure remained thermally stable. These findings support microfluidization as an effective strategy for developing stable flaxseed fiber-based nanoemulgels, with potential applications in functional food systems.

## 1. Introduction

The development of novel functional foods has gained great interest in recent years, driven by growing consumer demand for healthier and more natural products. In this context, oil-in-water (O/W) nanoemulgels have emerged as a versatile and promising tool in the design of structured foods as they combine the physicochemical properties of nanoemulsions with the texture and stability provided by gelled systems [1,2,3].

O/W nanoemulgels are characterized by nanometer-sized oil droplets dispersed in an aqueous phase that has been structured as a gel. This combination results in systems with a creamy appearance, high stability against phase separation, and modulable consistency [4]. In the food industry, such systems are explored as a basis for the development of products such as low-fat spreads, conventional fat substitutes, dressings, and others. They also represent an effective strategy to replace less healthy or synthetic ingredients with natural or fiber-rich components [5,6,7]. In this study, nanoemulgels were designed to emulate the characteristics of salad dressings—a widely consumed food product with significant market presence due to its popularity among consumers.

One of the aspects to be taken into account in the design of nanoemulgels for food use is the selection of natural, sustainable, and safe components, which not only structure the system but also provide functional and nutritional properties [8,9]. Flaxseed (*Linum usitatissimum*) fiber has attracted increasing interest as a plant-based ingredient, especially for its high contents of mucilage, soluble polysaccharides, lignans, and dietary fiber [10]. This fiber, obtained from the husk and part of the pericarp of flaxseed, not only has remarkable properties, such as the ability to form gels, retain water, and stabilize emulsions, but also offers nutritional benefits associated with digestive health, glucose and cholesterol regulation, and prebiotic function [11,12,13].

Flaxseed mucilage is mainly composed of galacturonic acid, xylose, rhamnose, and galactose, with arabinose, fucose, and glucose present in smaller amounts. Xylose is typically the dominant neutral sugar, and the exact monosaccharide composition varies depending on the extraction method [14]. Notably, flaxseed fiber is predominantly classified as a soluble dietary fiber owing to its physicochemical properties in aqueous environments, where it forms highly viscous solutions even at low concentrations [15]. This property makes it an excellent natural gelling agent. Flaxseed fiber also exhibits emulsifying and water/oil-binding properties. It exhibits non-Newtonian rheological behavior, typically pseudoplastic behavior [16], which means its use is favored in foods that require ease of application or consumption, such as spreads [12,17,18]. This fiber has been applied in oleogels, cryogels, aerogels, edible coatings, and as a stabilizer in emulsion systems [19]. In more applied contexts, flaxseed mucilage has been utilized as a stabilizer for model salad dressings [20] and has successfully functioned as a meat binder and as an additive in plant juices, dairy items, and flour products [21].

The application of flaxseed fiber in nanoemulgels represents a dual strategy: on the one hand, it improves the structure and stability of the system; on the other hand, it enriches the food with a functional ingredient rich in soluble fiber. This is particularly attractive for the development of products with nutritional claims such as being a ‘source of fiber’. Moreover, as a low-cost and high-yielding agro-industrial by-product, its use is in line with the principles of the circular economy and sustainability in the food industry [22,23].

The use of flaxseed fiber in nanostructured systems still requires further research. Factors such as variability in fiber composition and control of texture, flavor, or color are key aspects to be resolved for its implementation on an industrial scale. Rheological behavior and storage stability also need to be evaluated.

Various methods are available for the preparation of nanoemulgels, with one of the most commonly employed approaches involving a three-step procedure: (a) formulation and processing of the nanoemulsion, (b) preparation of the structured (gel) phase, and (c) subsequent mixing of both phases [24]. In contrast, the present study adopted a sequential, high-energy process that integrates rotor–stator homogenization followed by microfluidization. This methodological approach, combined with the use of dietary fiber—specifically flaxseed fiber—as a natural bio-stabilizer of a nanoemulgel mimicking salad dressing, represents the main innovations of this work. Moreover, based on an extensive review of the literature, no previous studies have systematically investigated the effect of the number of microfluidizer passes on the physicochemical and rheological properties of nanoemulgels formulated with this type of structure.

The aim of this work is to develop and characterize O/W nanoemulgels formulated with flaxseed fiber as a natural gelling agent. The effect of microfluidization cycles on the physical stability, particle size, and rheological properties of the system will be evaluated to determine optimal conditions. After determining the number of passes, the sample will be further analyzed for thermal stability through time sweeps and viscoelastic and flow tests. The results provide insights into the impact of microfluidization and temperature on the role of natural fibers as new bio-based stabilizers and offer a clean-label and plant-based alternative to traditional fat-based dressings.

## 2. Results and Discussion

### 2.1. The Impact of the Number of Passes Through the Microfluidizer

#### 2.1.1. Particle Size Distribution

**Figure 1:**
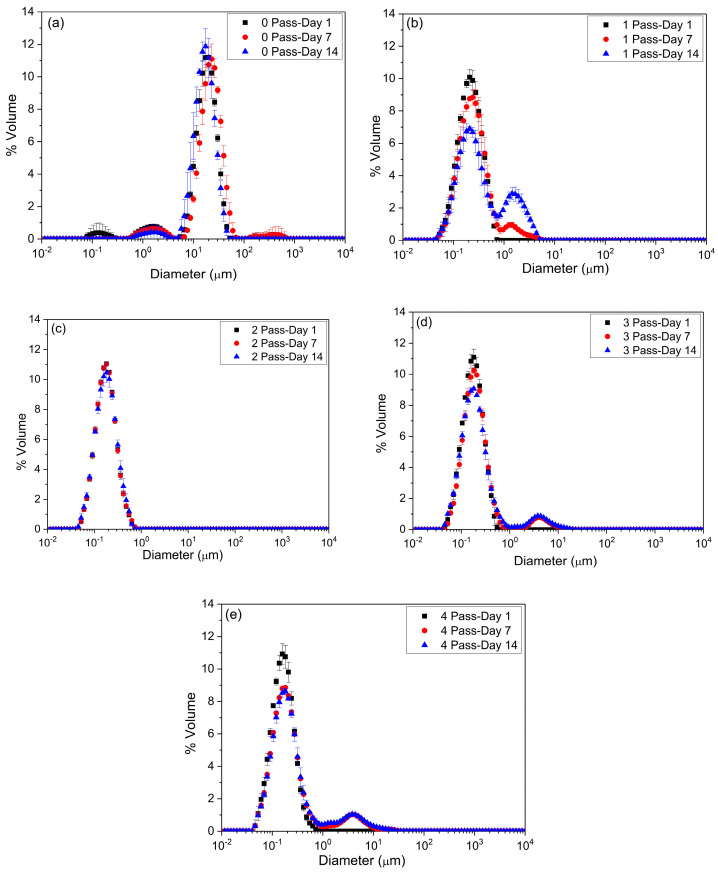
The effect of the number of passes through the microfluidization device on the droplet size distributions of flaxseed fiber emulgels evaluated at 1, 7, and 14 days. (**a**) Zero passes, (**b**) one pass, (**c**) two passes, (**d**) three passes, and (**e**) four passes. All measurements were conducted at room temperature. Data are presented as the mean ± standard deviation of a minimum of three replicates.

The determination of particle size and its distribution is of great importance in emulgels as it directly affects their stability and overall performance. Understanding the distribution patterns provides valuable insights into the effectiveness of the processing parameters applied. Figure 1 shows the particle size distribution of the emulgels as a function of the number of passes through the microfluidization device and the aging time (1, 7, and 14 days).

In the coarse emulgel, that is, the sample with zero passes (Figure 1a), the initial particle size distribution at day 1 was trimodal, with peaks centered approximately at 0.1 µm, 1 µm, and 10 µm. However, after 7 and 14 days of storage, the peak corresponding to the smallest size (~0.1 µm) disappeared, indicating possible coalescence or destabilization of the smaller droplets.

For the samples with one (Figure 1b), three (Figure 1d), and four passes (Figure 1e), the particle size distribution was initially monomodal (day 1), with a main peak around 0.1 µm indicating that no recoalescence by overprocessing occurred with an increase in the number of cycles, typical in microfluidization [25]. With aging (days 7 and 14), these emulgels developed a bimodal distribution, with a new peak around 1 µm becoming evident. The population of the greater particles increased, and the first peak decreased during aging. The shape of the particle size distribution as well as the fact that the oils used as a dispersed phase are hydrophobic rule out a destabilization mechanism by Ostwald ripening, suggesting that a coalescence phenomenon occurred [26]. This destabilization mechanism was particularly more pronounced in the one-pass sample, suggesting a lower stability versus time compared to the emulgels processed with more passes.

In contrast, the emulsion with 2 passes (Figure 1c) maintained a monomodal distribution centered at approximately 0.1 µm throughout the study period, indicating greater stability in terms of particle size. A similar distribution was found for nanoemulgels formulated with inulin and omega-3 fatty acid [1].

Table 1 shows the mean values of the Sauter diameter (D[3,2]), the mean volumetric diameter (D[4,3]), and the span dispersion parameter of the emulgels with 2.6 wt.% flaxseed fiber with aging time as a function of the number of passes during the step of microfluidization.

After 24 h of aging, a marked decrease is observed in both D[3,2] and D[4,3] from the coarse emulgel to the emulgel with one pass through the microfluidizer. In the untreated emulgel (0 passes), the mean diameters were significantly larger (6.25 ± 3.83 µm and 21.23 ± 7.57 µm, respectively). After a single pass, these values were drastically reduced to 0.17 ± 0.01 µm (D[3,2]) and 0.22 ± 0.01 µm (D[4,3]). It is worth highlighting the relevance of the low mean diameter values obtained after microfluidization, which were below 0.2 µm; this means that these emulgels can be considered as nanoemulgels After two passes, the diameter values continued to decrease slightly and stabilized around 0.13–0.15 µm for D[3,2] and around 0.17–0.18 µm for D[4,3], showing a saturation effect on homogenization efficiency with more than two passes. However, it should be noted that, from one to four passes, no significant differences in the mean diameters were found according to the ANOVA analysis. Llinares et al. (2021) [27] also found no significant decrease in the mean diameters with a plateau value (12–18 passes) by increasing the number of microfluidization cycles in nanoemulsions formulated with fennel oil. As for the span value, an indicator of particle size dispersion, a decreasing trend was observed with an increasing number of passes, remaining practically constant between two and four passes. This result suggests a more homogeneous particle size distribution as more mechanical energy is applied, but more than two passes do not significantly contribute to improving the uniformity of the system. In fact, the ANOVA analysis demonstrated that the span parameter was not sensitive to the number of recirculation cycles studied. Santos et al. (2021) [25] investigated the effect of microfluidization on zein-based emulsions and found an increase in the span parameter when increasing the number of passes. This result was explained by the overprocessing of the sample. However, this phenomenon, as mentioned above, did not occur in our samples, and for this reason, this processing variable is not significant.

Except for the nanoemulgel subjected to two passes, the aging time provokes a trend in which the mean diameters are increased, especially the D[4,3] diameter, which is more sensitive to particle aggregation. This result is in agreement with the destabilization process by coalescence observed in the evolution of the particle size distributions. However, regardless of the number of passes, except for the coarse emulgel, the aging time does not exert a statistically significant influence on the mean diameter values (Table 1), indicating good physical stability during the study period.

The obtained results suggest that an intermediate number of passes (in this case, two) could be optimal for obtaining a nanoemulgel with a homogeneous distribution and greater stability over time.

#### 2.1.2. Stress Sweep

This test is primarily conducted to identify the upper limit of the linear viscoelastic range (LVR), that is, the critical stress value, τ_c_. This stress, also known as the yield stress, provides valuable information on the fracture properties of the samples: lower values of τ_c_ indicate weaker samples that break more easily [16]. Provided that the applied stress remains below the critical value, the storage modulus (G′) and the loss modulus (G″) form a plateau that indicates that there are no significant structural changes in the sample under these low stresses or deformations. Therefore, the measurements carried out within the LVR zone are nondestructive tests.

**Figure 2:**
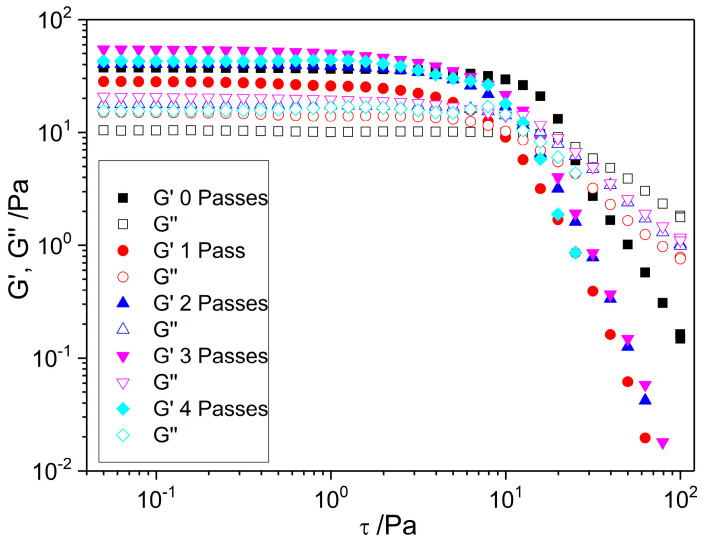
The influence of the number of passes through the microfluidization device on the mechanical spectra of nanoemulgels with 2.6 wt% flaxseed fiber. T = 20 °C.

Figure 2 presents the results of the stress amplitude sweeps performed on all samples, ranging from 0.05 Pa to 100 Pa at a fixed frequency of 1 Hz. The rheological response of the studied nanoemulgels shows that the G′ values increased by 88%, 126%, 162%, and 176% relative to G″ for one, two, three, and four passes, respectively, in the LVR, indicating a gel-like, solid behavior, that is, elastic behavior predominates viscous behavior, and the samples exhibit a certain rigidity [28]. Both moduli begin to decrease progressively beyond the upper limit of the LVR, except for the zero-pass emulgel, which exhibits a pseudo-dilatant peak in the loss modulus (G″). This behavior was also observed in certain gums [29], which was attributed to microstructural rearrangements under high stress.

There are several options to determine the LVR limit. In this work, it was visually established by drawing a straight line as a fitting line along the G′ and G″ plateaus. The most restrictive value (corresponding to the fitting line of G′ or the fitting line of G″) corresponds to the critical stress or yield stress. The analysis of the critical stress values obtained via the straight-line criterion leads to the conclusion that a number of passes ranging from one to four does not have an effect on the extension of the linear viscoelastic region since the τ_c_ values were found to be similar, approximately 2.5 Pa. The coarse emulgel exhibited a slightly higher value of this parameter, about 5 Pa.

From this test, it is also possible to determine the flow point (τ_f_), which corresponds to the stress value at which the gel-like behavior changes to the liquid state. It can be determined by employing the tangent criterion, that is, the stress corresponding to tan δ = 1 (G′ = G″). A clear reduction in this parameter occurred from the zero-pass emulgel to the one-pass sample (22.5 versus 8.7 Pa), but by increasing the number of passes, an increasing trend in the τ_f_ value was observed (11.7, 13.2, and 13.4 Pa for two, three, and four passes, respectively).

τ_f_ and τ_c_ allow for the flow transition index to be calculated, which is the τ_f/_τ_c_ ratio. It is a dimensionless ratio that helps to describe how the internal structure breaks under stress. Thus, as this value approaches 1, the brittleness of the sample increases. In our samples, the flow transition index decreased upon subjecting the coarse emulgel to microfluidization (from 4.5 to 3.5), indicating a more brittle structure. However, with an increasing number of microfluidization passes, this parameter rises progressively, further deviating from unity (reaching 4.7, 5.3, and 5.4 after two, three, and four passes, respectively). These results suggest that structural rearrangements occur with repeated processing, leading to a slight reduction in the brittleness of the resulting nanoemulgels. Nevertheless, the fact that the index values remain within the same order of magnitude indicates that these structural changes are not substantial.

#### 2.1.3. Frequency Sweep

**Figure 3:**
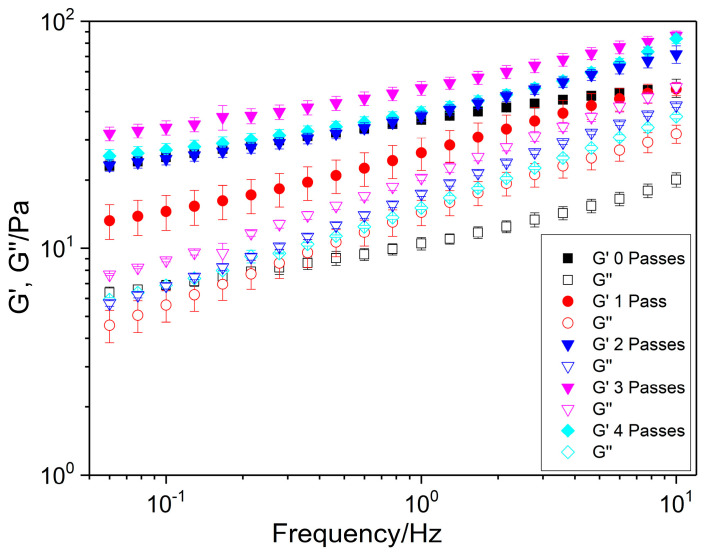
Frequency sweep of different nanoemulgels with 2.6 wt.% flaxseed fiber as a function of the number of passes through the microfluidizer.

The frequency sweeps provide valuable insights into the viscoelastic behavior of the systems and help to assess whether the internal structures are weakly or strongly associated. The rheological results obtained with this test are shown in Figure 3, where the storage modulus (G′) and the loss modulus (G″) of nanoemulgels containing flaxseed fiber are plotted as a function of the number of passes.

Across all the studied conditions, regardless of the number of passes applied (zero, one, two, three, and four passes), it can be clearly observed that the storage modulus, G′, consistently exceeds the loss modulus, G″, over the entire frequency range tested. This dominance of G′ over G″ indicates that the systems exhibit predominantly elastic behavior and a certain solid-like character. Nevertheless, the low values of both moduli and their frequency dependence suggest a weak internal network structure, typical of weakly associated systems [30,31,32]. According to Steffe (1996) [33], these nanoemulgels can be classified as weak gels because they exhibit the dependence of both moduli on the frequency. Furthermore, the viscoelastic moduli (G′ and G″) did not exhibit a crossover within the frequency range studied, indicating the absence of a gel-to-sol transition in the emulgel system [34]. This result is highly advantageous from a technological standpoint, particularly with regard to its application in food formulations. Comparable findings have been reported for other emulgels, such as emulgels formulated with soy protein isolate [35] or pectin-based emulgels [36] or emulgels stabilized with whey protein and xanthan gum [37]. To interpret this rheological behavior, it is instructive to consider analogous self-assembly and structural stabilization mechanisms from low-molecular-weight gelators described in non-food colloidal systems such as the Iridium (III) complex or alkylated benzimidazolone derivatives reported by Scarpelli et al. (2017) [38] and Makeiff et al. (2021) [39], respectively. In such systems, the gelation mechanism relies on non-covalent interactions, primarily hydrogen bonding and hydrophobic effects, to induce self-assembly into fibrillar networks that immobilize the solvent phase. Similarly, in our system, flaxseed fiber polysaccharides, with hydroxyl and carboxyl groups, contribute to network formation via physical entanglements and hydrogen bonding, resulting in the stabilization of dispersed oil droplets within a viscoelastic matrix.

Despite this general trend, a comparative analysis of the mechanical spectra revealed significant differences among the treatments, especially at low frequencies. A trend to increase the G′ value with the number of passes from one to three is observed. This result indicates that a more structured internal network and stronger intermolecular interactions are obtained by increasing the number of cycles. A similar result was found by Mccarthy et al. (2016) [40] and Yang et al. (2022) [41] when increased microfluidization pressure was placed on a pea protein-based emulgel or zein particle-based emulgels, respectively. Beyond 3 Pa, a decrease in G′ occurs as a consequence of an excess of input energy applied, which provokes a structural degradation and consequently a loss of microstructure [42]. Regarding the loss modulus, non-significant differences were found in its values for one, two, and four passes, exhibiting the highest value at three cycles.

These findings underscore the significant impact of the microfluidization intensity, expressed as the number of passes, on the viscoelastic properties of flaxseed fiber nanoemulgels. Understanding this relationship is critical for optimizing processing conditions in the development of nanoemulgel-based formulations, particularly in food, nutraceutical, and pharmaceutical applications where textural and stability properties are key to product performance.

#### 2.1.4. Physical Stability

**Figure 4:**
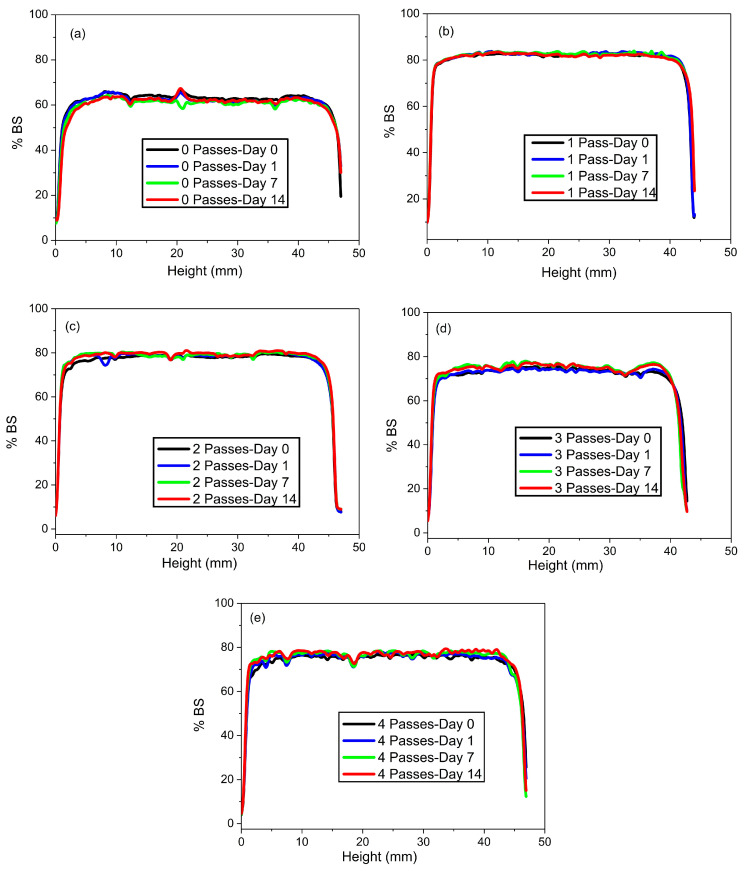
Variation in the backscattering percentage with the height of the vial containing flaxseed fiber nanoemulgels over time. (**a**) Zero passes, (**b**) one pass, (**c**) two passes, (**d**) three passes, and (**e**) four passes.

Figure 4 shows the stability analysis through the percentage of backscattered light as a function of storage time. It can be seen that all the formulations evaluated presented practically unchanged profiles over time, indicating remarkable physical stability.

**Figure 5:**
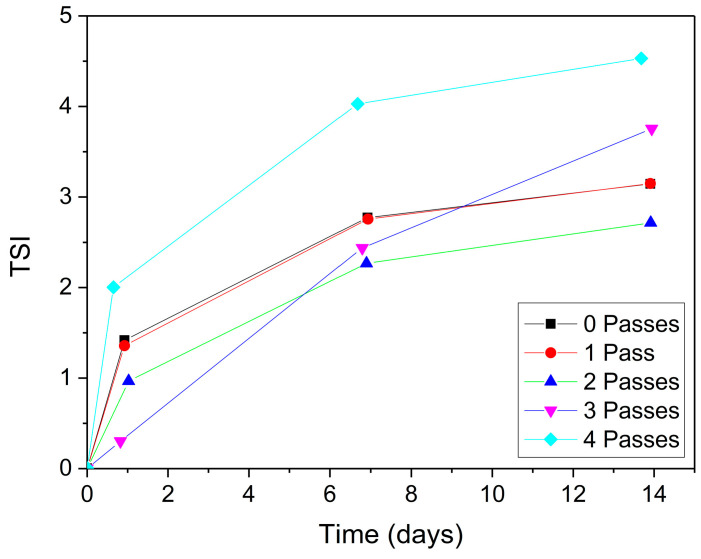
Changes in the Turbiscan Stability Index of flaxseed fiber nanoemulgels over the storage period.

To confirm these results, the Turbiscan Stability Index (TSI) values were calculated (Equation (7)) during the storage period, the results of which are presented in Figure 5. As can be seen, and in agreement with the backscattering profiles, all the samples studied can be considered stable, showing TSI values lower than 4, with the exception of the sample corresponding to four passes. It is important to highlight that the sample with two passes was the most stable, reaching a TSI value close to 2.

### 2.2. Influence of Temperature

Taking into account the results obtained previously, the sample processed with two passes was chosen, as it was the most stable, to study the influence of temperature on the rheological properties of this nanoemulgel.

#### 2.2.1. Time Sweep

**Figure 6:**
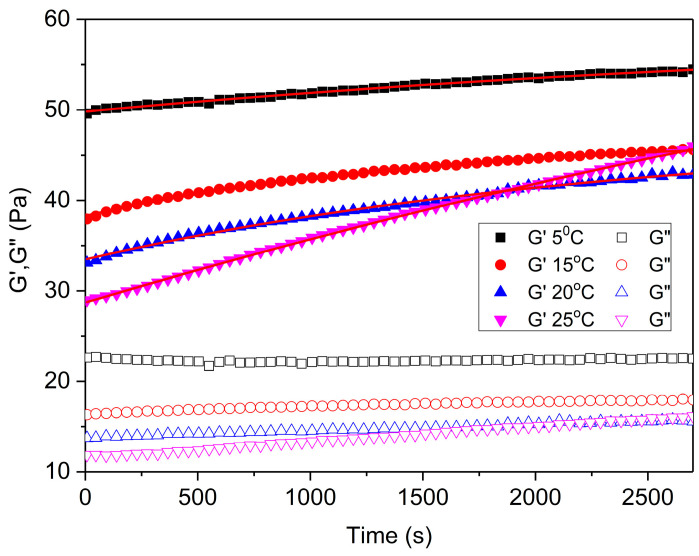
The effect of time on the storage modulus (G′) of the nanoemulgel with 2.6% flaxseed fiber with 2 passes in the M110P device as a function of temperature (5, 15, 20, and 25 °C). In addition, the fit of G′ to a first-order kinetic equation is shown.

The evolution of G′ at 1 Hz with resting time immediately after the sensor reaches the measuring position (1 mm) in the SAOS tests is shown in Figure 6 as a function of temperature. This type of test allows for the evaluation of the influence of the recent mechanical history, as evidenced by a progressive increase in the storage modulus with resting time until equilibrium values are attained. As shown in Figure 6, a marked decrease in the G′ values with increasing temperature is observed, which is most likely attributable to the enhanced molecular mobility induced by the Brownian motion and a concomitant reduction in the intermolecular interactions within both the fiber-based network structuring the continuous phase and the dispersed oil nanodroplets characteristic of the nanoemulgel system. This behavior indicates that the material acquires a more fluid nature at elevated temperatures, which translates into a lower capacity to store energy.

Within the temperature range of 5 °C to 20 °C, the time-dependent evolution of the storage modulus reached a steady-state value. However, a prolonged period is required to attain equilibrium. At 25 °C, a steeper slope is observed without reaching this equilibrium value compared to the other temperatures tested. This indicates that drying of the sample occurs from the beginning.

The recovery kinetics of G′, as previously reported by other authors [43,44], was fitted to a first-order kinetic equation (Equation (1)), and the fitting parameters are shown in Table 2. The ratio of $G_{0}^{\prime }$ to $G_{\infty }^{\prime }\;$decreased with increasing temperature as a result of the reduced structural resistance of the emulgel under deformation during the loading phase of the rheological measurement.

#### 2.2.2. Frequency Sweep

**Figure 7:**
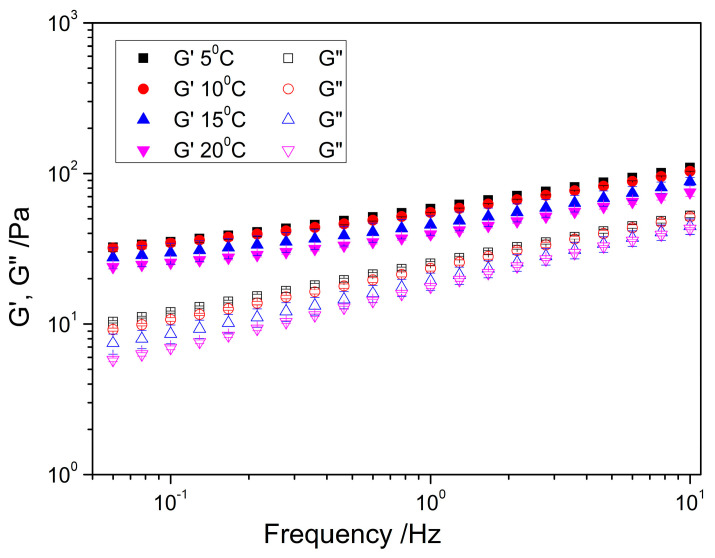
Frequency sweep of the nanoemulgel with 2.6% flaxseed fiber with 2 passes in the M110P device as a function of temperature (5, 10, 15, and 20 °C).

In all samples, viscoelastic behavior was observed throughout the frequency range analyzed, with a clear dominance of the elastic modulus (G′) over the viscous modulus (G″) (Figure 7). As observed at 1 Hz in the time sweeps, as the temperature increases, both moduli (G′ and G″) decrease, which is attributed to the loss of the structural stiffness of the material. This decrease implies a reduction in both the elasticity and viscosity of the system. However, it is worth noting that these changes are not significant.

Furthermore, the moduli exhibit a slight dependence on frequency. For G′, this dependence was well fitted to a power law equation (Equation (2)), which was previously used by other authors [45,46]. As shown in Table 3, when comparing the results obtained at different temperatures, it is observed that k tends to decrease and the slope *n* is practically constant. The results of the ANOVA test demonstrate that there were no significant differences in both parameters with temperature, suggesting a similar behavior in terms of the relative viscoelastic response, that is, this nanoemulgel exhibited a thermally stable microstructure in the temperature range studied (5–20 °C).

#### 2.2.3. Flow Curves

**Figure 8:**
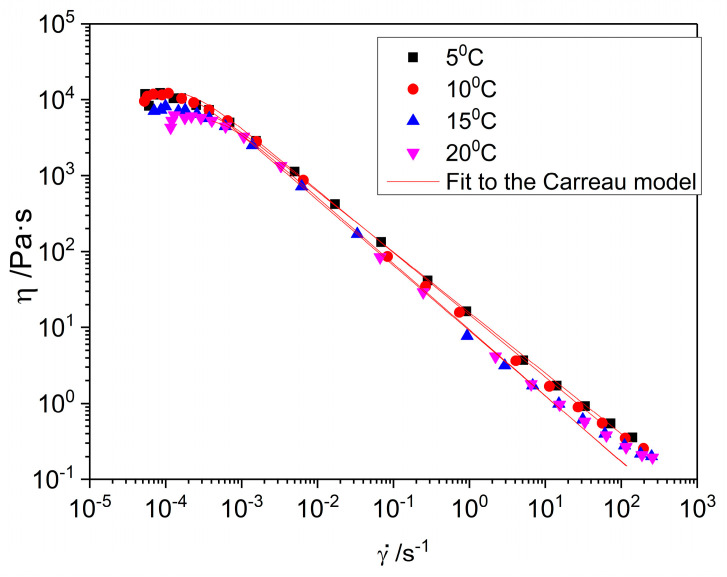
Flow curves of the nanoemulgel with 2.6% flaxseed fiber with 2 passes in the M110P device as a function of temperature (5, 10, 15, and 20 °C).

The flow curves of the nanoemulgel with 2.6% flaxseed fiber and two passes in the M110P device as a function of temperature are shown in Figure 8. At all temperatures studied, the sample exhibited a non-Newtonian shear-thinning response, with a plateau in viscosity at low shear rates corresponding to the zero-shear viscosity (η_0_). As the shear rate increased beyond a critical threshold ($\dot{\gamma_{c}}$), viscosity decreased following a power law trend. As the temperature increased, the viscosity of the nanoemulgel slightly decreased, which can be attributed to the weakening of the intermolecular interactions, mainly of the fiber polymer, and a slight structural breakdown of the system under thermal influence.

The flow curves were fitted to the Carreau equation [47] (Equation (3)), and the parameters obtained from the fit of the experimental data to the model are presented in Table 4. A slight tendency for η_0_ to increase with an increasing temperature is observed, which does not correspond with what is visually observed in Figure 8. Nevertheless, the analysis of the ANOVA indicates that these differences are not significant. In parallel, the values of the flow index, *n*, tend to decrease, indicating more pseudoplastic behavior at elevated temperatures. Consequently, the emulgel at 5 °C exhibited the lowest degree of shear-thinning behavior. This atypical rheological response may reflect the presence of structural heterogeneities, such as microaggregate formation, similar to what was previously suggested for pectin-based systems [48]. However, consistent with the behavior of the zero-shear viscosity, the observed variations are not considered statistically significant.

From the obtained results, it can be deduced that this nanoemulgel has good thermal stability in the range of 5 to 20 °C.

## 3. Conclusions

Emulgels designed to mimic salad dressings and structured with flaxseed fiber were developed through a two-step high-energy mechanical process involving in-series rotor–stator homogenization followed by microfluidization. The resulting emulgels exhibited monomodal particle size distributions with mean diameters below 220 nm (nanoemulgels) and demonstrated excellent physical and thermal stability, particularly the formulation processed with two microfluidizer passes. This formulation maintained consistent particle size and viscoelastic properties over time and temperature variations, indicating strong structural integrity. Rheological analysis revealed weak gel-like behavior, with greater viscoelastic moduli observed at three passes, beyond which overprocessing led to structural weakening. The robust thermal and physical stability, clean-label composition, and desirable rheological behavior make these flaxseed fiber-based nanoemulgels promising candidates for replacing traditional fat-based salad dressings in functional and plant-based food formulations. In addition, this study demonstrates that microfluidization is an effective method for producing stable nanoemulgels using natural structuring agents. Future research should focus on exploring the encapsulation of bioactive ingredients and their release kinetics and on conducting a longer-term physical stability study and sensory testing to determine consumers’ acceptance of the product, both of which are very interesting from a commercial perspective.

## 4. Materials and Methods

### 4.1. Materials and Sample Preparation

The nanoemulgel was obtained by preparing the aqueous and dispersed phases separately. First, the dispersed phase was prepared by adding 21 wt.% of a blend of chia oil (Bóveda, Lugo, Spain)/sunflower oil (Bela Vizago, Murcia, Spain) at 50 wt.% and 2.1 wt.% OSA starch N-Creamer 2111 (Ingredion, Westchester, NY, USA). They were mixed in IKA-Visc MR-D1 (Ika, Staufen, Germany) and a sawtooth-type impeller at 800 rpm for 30 min. The aqueous phase was prepared by adding 67 wt.% water, 5.8 wt.% white vinegar, 0.2 wt.% lemon juice, 0.75 wt.% salt, 0.1 wt.% potassium sorbate, and 2.6 wt.% flaxseed fiber (HiFood, Parma, Italy). To form the primary emulsion, the aqueous phase was mixed with the dispersed phase using an Ultraturrax T50 (Ika, Staufen, Germany) with the S50-G45F dispersion unit at 500 rpm for 40 s and at 4000 rpm for 1 min. Finally, the primary emulsion was submitted to high-pressure homogenization by microchannels using an M-110P microfluidizer (Microfluidics, based in Westwood, MA, USA) at 25.000 psi (138 MPa) with interaction chamber type F12Y (Y-type interaction chamber with a minimum internal dimension of 75 μm) and H30Z (Z-type interaction chamber with a minimum internal dimension of 200 μm) arranged in series. The outflow sample tube of the microfluidizer was cooled with water at 15 °C to minimize the increase in sample temperature. A 200 g sample was passed through the microfluidizer at the specified pressure (25,000 psi) for 1, 2, 3 or 4 cycles. After the preparation of the emulgels, they were maintained at 5 °C until characterization.

### 4.2. Characterization Methods

Rheological measurements were carried out on days 1, 7, and 14 after preparation. The oscillatory tests and the temperature sweeps were performed using an AR2000 controlled-stress rheometer (TA Instrument, New Castle, DE, USA) and a rough plate–plate geometry of 60 mm (gap = 1 mm). Stress sweeps were carried out from 0.05 to 100 Pa at a fixed frequency of 1 Hz, and frequency sweeps were carried out from 0.01 to 10 Hz. In these tests, the temperatures were varied at 5, 10, 15, and 20 °C. The temperature sweeps were performed at a frequency of 1 Hz for 45 min and at a stress located within the linear viscoelastic region. For the flow curves, the Haake Mars 40 controlled-stress rheometer (Thermo Fisher Scientific, Waltham, MA, USA) was used with an equilibration time of 5 min at a temperature ranging from 5 to 25 °C. All samples were run in duplicate, and the values are shown as the standard deviation of the two replicates.

The dependence of the storage modulus with time was fitted to a first-order kinetic equation:

(1) $$G^{\prime }=G_{0}^{\prime }+\left(G_{\infty }^{\prime }-G_{0}^{\prime }\right)\left[\left(1-exp{\left(-k\times t\right)}^{m}\right)\right]$$

where $G^{\prime }$ is the storage modulus, $G_{0}^{\prime }$ is the $G^{\prime }\;$instantaneous value for a zero recovery time as predicted by the model, $G_{\infty }^{\prime }$ is the $G^{\prime }$ value associated with full recovery, k is the kinetic coefficient, and m is a fitting parameter fixed at 1 according to first-order kinetics.

The storage modulus dependence with frequency was fitted to a power law equation:

(2) $$G^{\prime }=a\cdot \omega^{b}$$

where a reflects the magnitude of the storage modulus and b describes the extent of its frequency dependence.

The flow behavior was fitted to the Carreau model:

(3) η= η0 1+γ˙γc˙21−n2

where the fitting parameters are $\eta_{0},\;\dot{\gamma_{c}}$, and *n*. $\eta_{0}$ is the zero-shear viscosity (Pa·s), $\dot{\gamma_{c}}$ is the critical shear rate (s^−1^) (related to the onset of shear thinning behavior), and *n* is the flow index.

The particle size was measured using a Malvern Mastersizer 2000 laser diffraction instrument (Malvern, Panalytical Ltd., Malvern, UK). Measurements were made 1, 7, and 14 days after preparation.

The Sauter diameter (D[3,2]) and the volumetric diameter (D[4,3]) were used to analyze the data. The span was analyzed for polydispersity. The equations used are

(4) $$D\left\lfloor 3,2\right\rfloor =\frac{\sum_{i=1}^{N}n_{i}d_{i}^{3}}{\sum_{i=1}^{N}n_{i}d_{i}^{2}}$$

(5) $$D\left\lfloor 4,3\right\rfloor =\frac{\sum_{i=1}^{N}n_{i}d_{i}^{4}}{\sum_{i=1}^{N}n_{i}d_{i}^{3}}$$

where *N* represents the total number of particles, *d_i_* denotes the diameter of a particle, and *n_i_* is the number of particles with diameter *d_i_*.

(6) $$Span=\frac{\left(D\left[v,0.9\right]-D\left[v,0.1\right]\right)}{D[v,0.5]}$$

where *D*[*v*,0.9] and *D*[*v*,0.1] correspond to the 90th and 10th percentiles, respectively, and *D*[*v*,0.5] refers to the median. Each test was conducted three times at room temperature.

For physical stability, the samples were stored at 5 °C. It was determined by the multiple light scattering technique using Turbiscan Lab Expert (Formulation, Toulouse, France) equipment. The value of the Turbiscan Stability Index (TSI) parameter was calculated using Turbisoft 2.0.0.19 using the following equation:

(7) $$TSI=\frac{1}{N_{h}}\sum_{t_{min}}^{t_{max}}\sum_{z_{min}}^{z_{max}}\left|BS\left(t_{i},z_{i}\right)-BS\left(t_{i-1},z_{i}\right)\right|$$

where *t_max_* is the time, *t*, at which the TSI is calculated; *z_min_* and *z_max_* are the lower and upper selected height limits, respectively; *N_h_* is the number of height positions in the selected zone of the scan; and, therefore, TSI is the sum of all backscattering differences across the entire height of the measurement cell.

The data were statistically evaluated using an analysis of variance (ANOVA) in GraphPad Prism 8.0.2, followed by Tukey’s post hoc test to identify significant differences between means, with a significance threshold set at *p* < 0.05.

## Figures and Tables

**Table 1:** The effect of the number of passes through the microfluidization device on the mean Sauter diameter, volume-weighted diameter, and span values of the emulgel containing 2.6 wt% flaxseed fiber.

**Day 1**			
	**D[3,2] ± SD (μm)**	**D[4,3] ± SD (μm)**	**span ± SD**
0 Passes	6.25 ± 3.83 ^a,^*	21.23 ± 7.57 ^α,^*	1.45 ± 0.25 ^v,^*
1 Pass	0.17 ± 0.01 ^c,^*	0.22 ± 0.01 ^β,^*	1.43 ± 0.09 ^v,^*
2 Passes	0.15 ± 0.01 ^c,^*	0.18 ± 0.01 ^β,^*	1.35 ± 0.01 ^v,^*
3 Passes	0.14 ± 0.01 ^c,^*	0.17 ± 0.01 ^β,^*	1.30 ± 0.01 ^v,^*
4 Passes	0.13 ± 0.01 ^c,^*	0.17 ± 0.01 ^β,^*	1.33 ± 0.01 ^v,^*
**Day 7**			
0 Passes	9.91 ± 0.16 ^b,^*	28.23 ± 13.58 ^α,^*	1.45 ± 0.35 ^v,^*
1 Pass	0.19 ± 0.02 ^c,^*	0.35 ± 0.02 ^β,^*	2.06 ± 0.22 ^w,^*
2 Passes	0.15 ± 0.01 ^c,^*	0.19 ± 0.01 ^β,^*	1.37 ± 0.01 ^v,^*
3 Passes	0.16 ± 0.01 ^c,^*	0.47 ± 0.01 ^β,^*	1.75 ± 0.03 ^v,^*
4 Passes	0.15 ± 0.01 ^c,^*	0.69 ± 0.02 ^β,^*	7.08 ± 0.47 ^x,^*
**Day 14**			
0 Passes	10.85 ± 2.76 ^b,^*	16.28 ± 0.29 ^α,^*	1.22 ± 0.07 ^v,^*
1 Pass	0.22 ± 0.03 ^c,^*	0.62 ± 0.06 ^β,^*	6.57 ± 0.49 ^y,^*
2 Passes	0.15 ± 0.01 ^c,^*	0.19 ± 0.01 ^β,^*	1.43 ± 0.09 ^v,^*
3 Passes	0.15 ± 0.01 ^c,^*	0.55 ± 0.01 ^β,^*	2.64 ± 0.16 ^w,^*
4 Passes	0.16 ± 0.01 ^c,^*	0.73 ± 0.06 ^β,^*	9.50 ± 0.61 ^z,^*

* A statistically significant difference (*p* < 0.05) was observed based on Tukey’s test, considering the standard deviation. ^a^ and ^b^ indicate significant differences (*p* < 0.05) in D[3,2] in different groups; ^α^ and ^β^ indicate significant differences (*p* < 0.05) in D[4,3] in different groups. ^c^, ^v^, ^w^, ^x^–^z^ indicate significant differences (*p* < 0.05) in the span values of different groups.

**Table 2:** Fitting parameters of the first-order kinetic equation applied to the nanoemulgel with 2.6% flaxseed fiber with 2 passes in the M110P device as a function of temperature (5, 15, 20, and 25 °C).

Temperature	$G_{0}^{\prime }$ (Pa) ± SD	($G_{\infty }^{\prime }\;-G_{0}^{\prime }$) (Pa) ± SD	k ± SD	R^2^
G′/5 °C	49.8 ± 0.03 ^a,^*	9.5 ± 0.09 ^c,^*	2.5×10^−4^ ± 0.08 ^e,^*	0.99
G′/10 °C	38.3 ± 0.05 ^a,^*	8.8 ± 0.11 ^c,^*	6.4×10^−4^ ± 2.02·10^−5 e,^*	0.99
G′/15 °C	33.5 ± 0.07 ^a,^*	14.2 ± 0.45 ^c,^*	6.4×10^−4^ ± 2.40·10^−5 e,^*	0.99
G′/20 °C	28.7 ± 0.04 ^b,^*	56.5 ± 2.69 ^d,^*	1.3×10^−4^ ± 7.59·10^−6 e,^*	0.99

* A statistically significant difference (*p* < 0.05) was observed based on Tukey’s test, considering the standard deviation. ^a^ and ^b^ indicate significant differences (*p* < 0.05) in the G′_0_ value of different groups; ^c^ and ^d^ indicate significant differences (*p* < 0.05) in $(G_{\infty }^{\prime }-G_{0}^{\prime })$ in different groups. ^e^ indicates significant differences (*p* < 0.05) in the *n* of different groups.

**Table 3:** Fitting parameters of the experimental data to the power law equation at the different temperatures studied. K is the y-intercept and *n* is the slope.

Temperature	K ± SD (Pa)	*n* ± SD	R^2^
G′/5 °C	1.76 ± 4.89×10^−4 x,^*	0.27 ± 6.76×10^−4 α,^*	0.99
G′/10 °C	1.73 ± 8.57×10^−4 x,^*	0.27 ± 1.00×10^−3 α,^*	0.99
G′/15 °C	1.64 ± 1.00×10^−3 x,^*	0.29 ± 2.00×10^−3 α,^*	0.99
G′/20 °C	1.59 ± 4.99×10^−4 x,^*	0.28 ± 8.05×10^−4 α,^*	0.99

* A statistically significant difference (*p* < 0.05) was observed based on Tukey’s test, considering the standard deviation. ^x^ indicates significant differences (*p* < 0.05) in the y-intercept (K) of different groups; ^α^ indicates significant differences (*p* < 0.05) in the slope (*n*) of different groups.

**Table 4:** Fitting parameters for the Carreau model of the two-pass samples at different temperatures.

Temperature	η_0_ ± SD (Pa·s)	$\dot{\gamma_{c}}$ ± SD (s^−1^)	*n*	R^2^
5 °C	11,103.7 ± 286.02 ^a,^*	4.01×10^−4^ ± 5.29×10^−6 α,^*	0.21 ^x,^*	0.99
10 °C	11,760.33 ± 1508.68 ^a,^*	2.98×10^−4^ ± 3.25×10^−5 α,^*	0.16 ^x,^*	0.99
15 °C	11,494.9 ± 302.53 ^a,^*	1.46×10^−4^ ± 4.55×10^−5 α,^*	0.15 ^x,^*	0.99
20 °C	12,659.97 ± 340.03 ^a,^*	2.66×10^−4^ ± 4.10×10^−5 α,^*	0.13 ^x,^*	0.99

* A statistically significant difference (*p* < 0.05) was observed based on Tukey’s test, considering the standard deviation. ^a^ indicates significant differences (*p* < 0.05) in the η_0_ value of different groups; ^α^ indicates significant differences (*p* < 0.05) in the $\dot{\gamma_{c}}$ value of different groups. ^x^ indicates significant differences (*p* < 0.05) in the *n* of different groups.

## Data Availability

The data presented in this study are available upon request from the corresponding author.
